# The Nod-Like Receptor (NLR) Family: A Tale of Similarities and Differences

**DOI:** 10.1371/journal.pone.0002119

**Published:** 2008-04-30

**Authors:** Martina Proell, Stefan J. Riedl, Jörg H. Fritz, Ana M. Rojas, Robert Schwarzenbacher

**Affiliations:** 1 Department of Molecular Biology, University of Salzburg, Salzburg, Austria; 2 The Burnham Institute for Medical Research, La Jolla, California, United States of America; 3 Department of Immunology, University of Toronto, Toronto, Ontario, Canada; 4 Structural Computational Biology Group, Spanish National Cancer Research Center, Madrid, Spain; University of Cambridge, United Kingdom

## Abstract

Innate immunity represents an important system with a variety of vital processes at the core of many diseases. In recent years, the central role of the Nod-like receptor (NLR) protein family became increasingly appreciated in innate immune responses. NLRs are classified as part of the signal transduction ATPases with numerous domains (STAND) clade within the AAA+ ATPase family. They typically feature an N-terminal effector domain, a central nucleotide-binding domain (NACHT) and a C-terminal ligand-binding region that is composed of several leucine-rich repeats (LRRs). NLRs are believed to initiate or regulate host defense pathways through formation of signaling platforms that subsequently trigger the activation of inflammatory caspases and NF-kB. Despite their fundamental role in orchestrating key pathways in innate immunity, their mode of action in molecular terms remains largely unknown. Here we present the first comprehensive sequence and structure modeling analysis of NLR proteins, revealing that NLRs posses a domain architecture similar to the apoptotic initiator protein Apaf-1. Apaf-1 performs its cellular function by the formation of a heptameric platform, dubbed apoptosome, ultimately triggering the controlled demise of the affected cell. The mechanism of apoptosome formation by Apaf-1 potentially offers insight into the activation mechanisms of NLR proteins. Multiple sequence alignment analysis and homology modeling revealed Apaf-1-like structural features in most members of the NLR family, suggesting a similar biochemical behaviour in catalytic activity and oligomerization. Evolutionary tree comparisons substantiate the conservation of characteristic functional regions within the NLR family and are in good agreement with domain distributions found in distinct NLRs. Importantly, the analysis of LRR domains reveals surprisingly low conservation levels among putative ligand-binding motifs. The same is true for the effector domains exhibiting distinct interfaces ensuring specific interactions with downstream target proteins. All together these factors suggest specific biological functions for individual NLRs.

## Introduction

Eukaryotes have evolved complex systems to detect microbial infection and other potential threats to the host. Recognition of microbes relies on the sensing of microbe associated molecular patterns (MAMPs) by germline-encoded host pattern recognition molecules (PRMs), which include various families of leucine-rich repeat (LRR) bearing proteins in plants and animals. While Toll-like receptors (TLRs) constitute the main sensors for detection of extracellular microbes, recent findings suggest that two distinct protein families, the RIG-like helicases (RLHs) and the Nod-like receptors (NLRs), act as intracellular surveillance molecules [1]–[3]. Several proteins of the highly conserved NLR family have been shown to function as intracellular PRMs for the initiation of innate and adaptive immune responses upon pattern-specific sensing of microbes [4].

Like TLRs, NLRs are thought to recognize microbial products, as well as other intracellular danger signals, thereby initiating host defense pathways through the activation of the NF-kB response and inflammatory caspases [5]. Moreover, the NLR family has gained increased attention, since polymorphisms in certain NLR genes are linked to inflammatory disorders such as Blau syndrome, Crohn's disease or early-onset sarcoidosis [6].

Structurally, NLRs are large multi-domain proteins with a tripartite architecture. NLR proteins typically contain a central nucleotide-binding domain termed NACHT domain (often also referred to as NOD domain), N-terminal effector domains (PYRIN, caspase recruitment domain CARD, or baculovirus inhibitior of apoptosis protein repeat BIR domain) for binding downstream signaling molecules, while the C-terminal part consists of a receptor domain, which is characterized by a series of leucine-rich repeats (LRRs). It is hypothesized that the crucial step in NLR activation lies in the oligomerization of the NACHT-domain, thereby forming an active signaling platform (e.g. the inflammasome or nodosome [7], [8], respectively), which allows binding of adaptor molecules and effector proteins, ultimately leading to an inflammatory response.

To date, 22 members of the human NLR protein family have been reported, which can be distinguished depending on the presence of a PYRIN, CARD, BIR, and a yet unclassified effector domain (Table 1).

**Table 1:** Overview of NLR family members according to their domain organization.

Nomenclature	Synonyms	Domain structure	Chrom. location	Genebank
***CARD domain***				
NOD1	**NLRC1**,CARD4	CARD-NACHT-WH-SH-LRR	7p14.3	AF126484
NOD2	**NLRC2**, CARD15	CARD-CARD-NACHT-WH-SH-LRR	16q12.1	AF178930
CIITA type1	**NLRA**	CARD-(X-NACHT-WH-SH-LRR)	16p13.13	AF000002
***PYRIN domain***				
NALP1	NLRP1, CARD7	PYD-NACHT-WH-SH-LRR-FIIND-CARD	17p13.2	AB023143
NALP2	**NLRP2**, Pypaf2	PYD-NACHT-WH-SH-LRR	19q13.42	AK000517
NALP3	**NLRP3**, Pypaf1	PYD-NACHT-WH-SH-LRR	1q44	AF054176
NALP4	**NLRP4**, Pypaf4	PYD-NACHT-WH-SH-LRR	19q13.43	AF479747
NALP5	**NLRP5**, NOD14, Pypaf8	PYD-NACHT-WH-SH-LRR	19q13.43	AY154460
NALP6	**NLRP6**, Pypaf5	PYD-NACHT-WH-SH-LRR	11p15.5	AF479748
NALP7	**NLRP7**, NOD12, Pypaf3	PYD-NACHT-WH-SH-LRR	19q13.42	AF464765
NALP8	**NLRP8**, NOD16	PYD-NACHT-WH-SH-LRR	19q13.43	AY154463
NALP9	**NLRP9**, NOD6	PYD-NACHT-WH-SH-LRR	19q13.43	AY154464
NALP10	**NLRP10**, NOD8, Pynod	PYD-NACHT-WH-SH	11p15.4	AY154465
NALP11	**NLRP11**, NOD17, Pypaf6	PYD-NACHT-WH-SH-LRR	19q13.42	AY095145
NALP12	**NLRP12**, Pypaf7, RNO	PYD-NACHT-WH-SH-LRR	19q13.42	AY095146
NALP13	**NLRP13**, NOD14	PYD-NACHT-WH-SH-LRR	19q13.42	AY154468
NALP14	**NLRP14**	PYD-NACHT-WH-SH-LRR	11p15.4	BK001107
***BIR domain***				
NAIP	**NLRB1**, BIRC1	BIR-NACHT-WH-SH-LRR	5q13.1	U19251
***untypical CARD***				
Ipaf	**NLRC4**, Card12, Clan	Card?-NACHT-WH-SH-LRR	2p22-p21	AF376061
NOD4	**NLRC5**, NOD27	Card?-NACHT-WH-SH-LRR	16q13	AF389420
NOD3	**NLRC3**	Card?-NACHT-WH-SH-LRR	16p13.3	BK001112
***undefined***				
NOD5	**NLRX1**, NOD26, NOD9	X-NACHT-WH-SH-LRR	11q23.3	AB094095
CIITA	**NLRA**	X-NACHT-WH-SH-LRR	16p13.13	U18259

Protein name and synonyms (new synonyms in bold), accession number, and chromosomal location according to http://www.genenames.org/genefamily/nlr.php followed by domains as defined by FFAS. CARD, caspase activation and recruitment domain; PYD, pyrin; NACHT, domain present in NAIP, CIITA, HET-E, TP-1; NALP, NACHT-LRR-PYD-containing protein; WH, winged helix domain; SH, superhelical domain, LRR, leucine-rich repeats.

According to the current general paradigm, NLR signaling is believed to be initiated by the C-terminal LRR region through the recognition of molecules triggering NLR activation. However, the actual molecular switch, namely the oligomerization of the NLR, then is thought to be mediated by the NACHT domain in a nucleotide-dependent manner. Recent studies show that Ipaf [9] and NALP3 [10] selectively bind ATP/dATP and that nucleotide binding is essential for their function in downstream signaling. Once the switch has occurred, the signal is transferred to the effector proteins such as inflammatory caspases or adaptor molecules, via their effector domains. Thus, CARD-containing NLRs such as NOD1 and NOD2 are thought to interact with the CARD-containing kinase RICK (RIP2) leading to the activation of CARD9 and NF-κB pathways [11]. In contrast, several PYRIN domain containing Nalp proteins were found to form a signaling platform, dubbed inflammasome, and drive caspase-activation by binding to the adaptor protein ASC [1], [7], [12], [13].

Despite the growing amount of research data, little is known about the precise molecular mechanism of NLR activation and the initiation of subsequent signaling cascades. Moreover, the structural and mechanistic data on NLR proteins is scarce and mainly limited to single effector domains. Recent studies by Albrecht *et al* discussed models of the NACHT and LRR domains of NOD2 and NALP3 in relation to disease associated SNPs and protein function [14]. Here, we provide further insights into structural and functional relationships of NLRs based on detailed sequence and modeling analyses of the whole NLR family. We show that although Apaf-1 shares less than 15% sequence identity to most NLRs and contains a different receptor domain (WD40 repeats), its structure, mode of action, and mechanistic principles can serve as a valuable working model for NLR signaling. In addition, we investigated the N-terminal effector domains (CARD and PYRIN) of the NLR protein family to construct a prediction for potential interfaces and interacting partners. Furthermore, we analyzed sequences of the LRR domains for conserved regions that may play a putative role in ligand binding and/or interaction with the NACHT and effector domains. Finally, we created a homology model of NOD2 based on Apaf-1 and the ribonuclease inhibitor (pdb id: 1dfj), which we used to depict disease related polymorphisms and mutations [6].

## Results and Discussion

### 1.1 NLR domain structure

**Figure 1:**
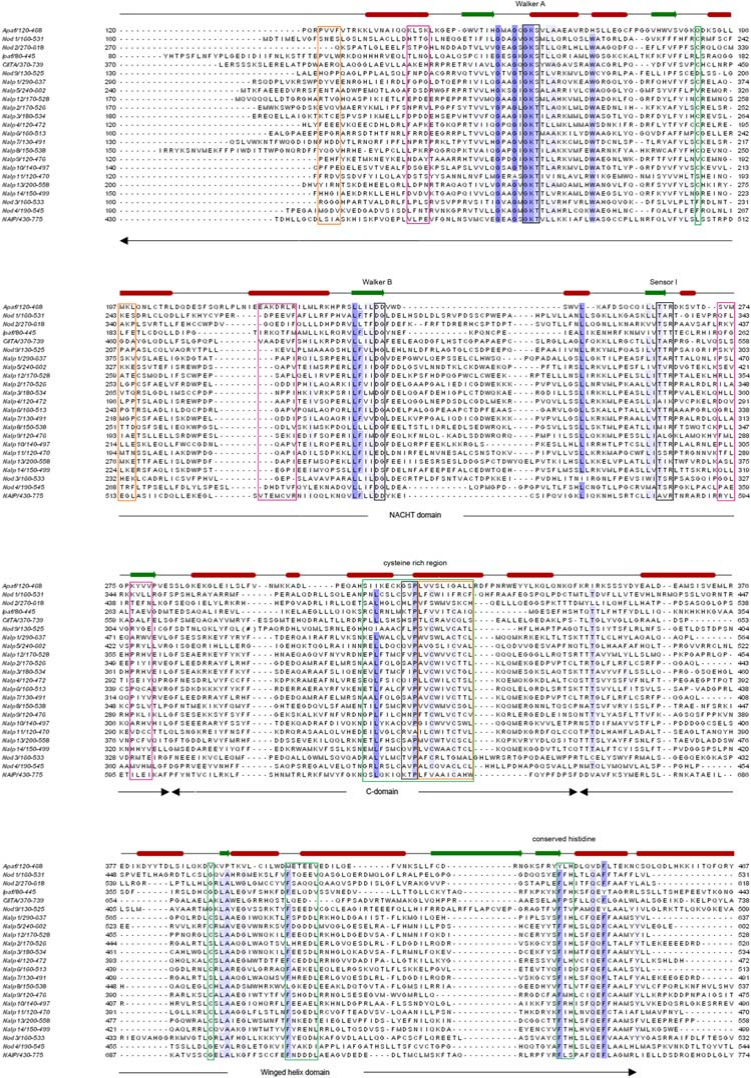
Multiple sequence alignment of NLR NACHT-WH-SH domains and the Apaf-1 NACHT-WH-SH domain. Degree of conservation is shown as blue shading. The secondary structure of Apaf-1 is shown above the Apaf-1 sequence. Arrows underneath the alignment indicate domain boundaries. Conserved sequence features important for catalytic activity are shown in black boxes. Orange and magenta boxes depict interfaces residues while the orange ones contribute to interactions with the left partner and the magenta residues are thought to interact with the right partner in the oligomer. Green boxes indicate additional motifs as described in the text.

To elucidate relations of NLR proteins, we used NLR sequences (see Table 1) as separate queries for FFAS searches [15] and secondary structure prediction with the PredictProtein Server [16]. Furthermore, a domain profile search using Interpro and SMART [17] was performed to verify the domain structure of individual NLRs. Comparative sequence analyses (Figure 1, Table 2) revealed that all NLR proteins belong to the AAA+ ATPase superfamily [18] where they are further classified as signal transduction ATPases with numerous domains (STAND) [19]. The STAND proteins are distinguished from other P-loop NTPases by the presence of unique sequence motifs associated with the N-terminal helix and the core β-strand-4, as well as a C-terminal helical bundle that is fused to the NTPase domain [19].

**Table 2:** Key elements of Apaf-1 and NLR NACHT-WH-SH domains extracted from the multiple sequence alignment in Figure 1.

	Seq-ID	Walker A	Walker B	Sensor 1	WH His	WH cons
**Apaf-1**	**-**	GKS	LLIL**DD**VWDSW	TTR	H438	**METEEV**
**NOD1**	9%	GKS	LFTF**DG**LDELH	TAR	H517	FTQEEV
**NOD2**	8%	GKS	LLTF**DG**FDEFK	TSR	H603	FSAQQL
**Ipaf**	9%	GKS	LFLL**DG**YNEFK	TTT	H443	FELQDV
**NALP1**	11%	GKS	LFIL**DG**VDEPG	TAR	H623	FSPDDL
**NOD5**	12%	GKS	LFVL**HG**LEHLN	TTR	-	FSEEDV
**CIITA**	11%	GKS	LLIL**DA**FEELE	TAR	-	LQED--
**NALP5**	10%	GKS	LFII**DG**FDDLG	TVR	H584	FDGDDL
**NALP12**	11%	GKS	LFII**DG**FDELK	TTR	H514	FEEQDL
**NALP6**	12%	GKT	LFIL**DG**ADELP	TTR	-	FAEKEL
**NALP7**	10%	GKT	LFVV**DG**LDELK	TTR	H468	FHREDL
**NALP8**	9%	GKT	LLLL**DG**FEELT	MIR	-	LGKEDL
**NALP9**	8%	GKT	LFIM**DG**FEQLK	ALG	H445	FSHGDL
**NALP10**	13%	GKT	LFIL**DG**FDELQ	TTR	H466	FEEAEL
**NALP13**	9%	GKT	LFII**DG**FEEII	TIK	H536	FNKEDT
**NALP14**	10%	GKT	LFII**DS**FDELN	TTR	H480	FYRENL
**NALP11**	9%	GKT	LFIL**ED**LDNIR	SSR	H450	FSGEDL
**NAIP**	14%	GKT	LFLL**DD**YKEIC	AVR	-	FNDDDL
**NOD3**	12%	GKT	LLIL**DG**LDECR	TSR	H502	FYEQDM
**NOD4**	8%	GKT	LLIF**DG**LDEAL	TSR	H491	FYAKDI
**NALP2**	10%	GKT	LFVI**DG**FDELG	TTR	H503	LHREDL
**NALP3**	10%	GKT	LFLM**DG**FDELQ	TTR	H520	FEESDL
**NALP4**	10%	GKT	LFVI**DS**FEELQ	AIK	H452	FCEDDL

Sequence identities shown are derived from pairwise alignments using FFAS between Apaf-1 and individual NLRs. WH His and WH cons show the conserved histidine and the conserved METEEV sequence patch, respectively which are located in the winged helix (WH) domain.

**Figure 2:**
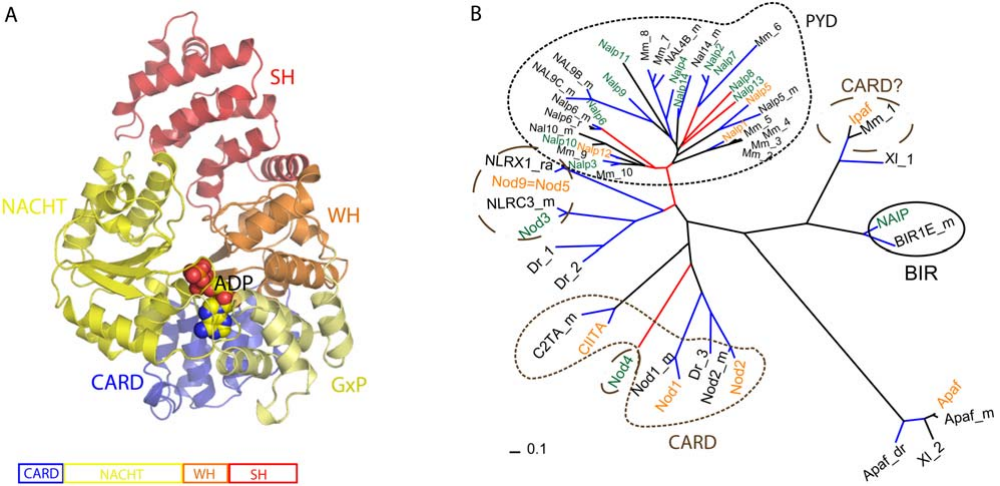
A Apaf-1 structure and domain organization. Apaf-1ΔWD40 (PDB id: 1z6t) is shown in ribbon representation color-coded according to domain boundaries (CARD aa 1–101, NACHT-GxP aa108–365, WH aa366–450, and SH aa451–586. ADP molecule bound to the active site is shown in sphere representation. B Evolutionary tree of human NLR NACHT-WH domain generated for NLR proteins and orthologues. Labels correspond to the accession numbers (Uniprot) Mm_1: Q3TAU8, Mm_2: Q2LKU8, Mm_3: Q2LKU9, Mm_4: Q2LKV8, Mm_5: A1Z198; NALP5_m: NALP5_MOUSE, Mm_6: Q4PLSO, Mm_7: Q66JP4; Mm_8: NAL4C_MOUSE, Mm_9:Q08EE9, Mm_10: Q8R4B8. Dr_1: A5PEZ1; Dr_2: A3KQD4, Dr_3: Q1AMZ9, Xl_1: Q28DS5, Xl_2: Q6GNU6. Black lines indicate a probability of more than 50%, blue lines indicate a 95% confidence in the grouping, and red lines indicate a confidence below the 40%. Green fonts indicate human proteins, black fonts alternative organisms. Mm and _m is *Mus musculus* (mouse), Dr and _Dr is *Danio rerio* (fish), Xl and _Xl is *Xenopus leavis* (frog). Orange fonts indicate an “S” in the Walker A motif in human sequences. Circles and modified ovals over the clades indicate the type of domain present at the N-terminal region of the NACHT domain. PYD is Pyrin domain, CARD is Card domain, Card? indicates CARD-like and BIR are BIR repeats. Apaf-1 is used to root the tree.

A sequence profile based search using FFAS identifies Apaf-1 as a distantly related homologue of NLRs within the human genome. The main difference between Apaf-1 and NLRs is the lack of a LRR domain. Instead, Apaf-1 utilizes two sets of WD40 repeats as receptor domain for sensing cytochrome c as specific trigger of apoptosis [20]. However, despite the different receptor domain, the remainder of Apaf-1 (residues 1–581) aligns with a sequence identity of 10–15% and a FFAS score of −16 to members of the NLR family. The significance of this sequence alignment is highlighted by the FFAS score of −16, which indicates high structural similarity despite the low sequence identity [15]. Apaf-1_1–581_ is also the closest hit among structurally characterized homologues, and therefore was chosen for homology modelling to decipher the mechanistic and structural features defining organization and function of NLR family members. Despite the existence of small differences in the ATPase domain of Apaf-1 and NLRs, our detailed secondary structure comparison and alignment analysis show that they share a common domain structure (Table 1), consisting of an N-terminal effector domain, a central NACHT domain, a winged helix (WH) domain, and a superhelical (SH) domain, followed by a LRR receptor domain of variable length (Figure 2A).

Variations thereof lie mainly within the effector domains, additional domains and within the length of the linker from the effector domain to the NACHT domain. Good examples for this are NOD2, which contains two N-terminal CARD domains, NOD5 and CIITA, which show an undefined N-terminal region. Other examples are the untypical secondary structure prediction for the CARD domain of Ipaf, or the partial sequence of the type 1 isoform of CIITA (accession number: AF000002) [21], which contains an alternative 5′ region that encodes a CARD domain.

In respect to the C-terminal LRR region NALP10 is the only NLR member that has no or only a very short occurrence of LRR repeats. NALP1 on the other hand shows a typical LRR region, but contains two additional domains C-terminally of those LRRs; a FIIND domain of yet unknown function and a C-terminal CARD domain (Table 1) that displays the typical secondary structure found in the N-terminal effector CARD domains of other NLRs. As outlined above and despite the low sequence identity the structure of Apaf-1 can be used for homology modeling purposes to obtain insight into the mechanism of NLR function and furthermore to produce approximate models of NLR structures.

### 1.2 NLR evolutionary profiles

Detailed sequence comparisons of 22 human NLR members reveal an overall sequence identity in the range of 10–30% amongst pairs. Since domain shuffling is a eukaryotic hallmark and has created a large complexity of functions in proteins, it hampers evolutionary analysis of full length proteins. In particular effector domains are subject to domain accretion, and/or domain shuffling or duplication for the acquisition of new domain architecture. Taking this into account, we have chosen the NACHT domain to conduct a phylogenetic analysis, addressing the possible evolutionary history of NLR proteins.

By comparing the NACHT region of the NLR family members, we observed that the phylogenetic distribution clearly correlates with their respective effector domain composition (Figure 2B). For instance, all the PYD-NACHT containing proteins clade together at the highest part of the tree, well separated from other domain combinations such as CARD-NACHT. In humans, these PYD-NACHT containing proteins have been expanded by several duplication events. Similar results were obtained by including other NLR sequences of non-human origin, demonstrating a clear distribution in agreement with the effector domain content. Moreover, 9 out of 14 proteins (NALP2, 4, 5, 7, 8, 9, 11, 12, 13) are located at chromosome 19 and clustered very closely together, which indicates a major expansion of this genomic region. Three other members (NALP6, NALP10 and NALP14) are located at chromosome 11, whereas NALP1 and NALP3 are located at chromosomes 17 and 1, respectively. Thus, we further analyzed whether these proteins have corresponding orthologues in closely related organisms. Clear orthologues were found for all proteins with the exception of NALP8, NALP11, NALP13 and NOD4. In addition we observed that NALP2 and NALP7 are recent duplicons within the human genome. For other organisms however, the expansion of this family originated from different members (data not shown), suggesting that NLRs of non-human origin have been lost during evolution. Moreover, these observations point to the possibility that the development for human paralogues reflects a way to accommodate novel functions to match the complexity of innate immunity in highly developed organisms.

### 1.3 The NLR NACHT-WH-SH region shows distinct adaptions in NLR function

Members of the AAA+ superfamily feature a so called ATPase, P-loop or Rossman-fold which adopts a three-layered α-β sandwich configuration. This fold contains recurring regulatory units with the β-strands forming a central, parallel β-sheet, which is embedded between α-helices on both sides (scop id C.37.1.20). The parallel β-sheet forming the core of the ATPase domain assumes a 51432 topology [22]. This fold contains several characteristic motifs, namely the Walker A/P-loop and Walker B motif, and the Sensor 1 and Sensor 2 motif. These motifs are involved in ATP-binding and hydrolysis of the β-γ phosphate diester bond [19] leading to specific conformational changes.

**Figure 3:**
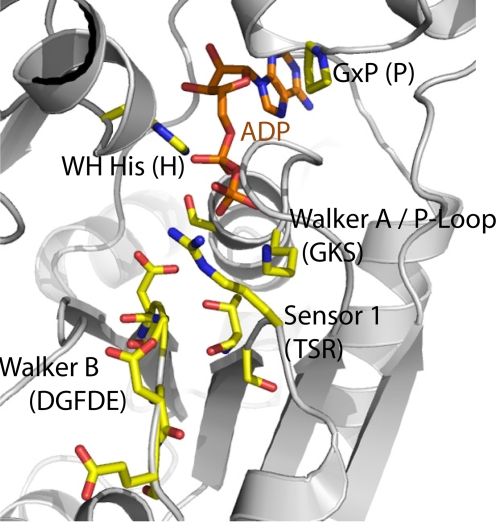
Model of the NOD2 nucleotide-binding site with an ADP molecule and conserved sequence motifs Walker A, Walker B, Sensor 1, GxP, and WH-His shown in sticks.

To decipher the structure and mechanism of the NLR protein family we performed a multiple sequence alignment of NLR proteins and Apaf-1 (Figure 1) focusing on the NACHT-WH-SH domains using the program muscle [23]. The outcome was then compared with FFAS [15] search results. Subsequently, we utilized secondary structure prediction and homology modelling (Figure 3) to decipher the presence of critical ATPase motifs like Walker A/P-loop, Walker B, Sensor 1 and Sensor 2 to deduce putative functional features unique to NLRs. As shown in the multiple sequence alignment (Figure 1), the overall secondary structure features of the NACHT domain are conserved among NLR proteins and Apaf-1 (Table 2). We observed that the only main difference is constituted by a deletion before β-strand 3 and a 20 residue insertion after β-strand 3 in the NACHT domain.

**The Walker A** motif is composed of the characteristic consensus pattern GxxxxGKT/S (x represents any amino acid), where the lysine residue directly interacts with a phosphate moiety of ATP [24]. We observed that based on the presence of a threonine or serine residue in the GKT/S sequence motif, members of the NLR protein family can be subdivided into two groups (Table 2). This separation is reflected in the evolutionary tree (Figure 2B, where orange fonts indicate a presence of S instead of T) in which the phylogenetic distribution follows the T/S signature. Then, NALP1, NALP5 and NALP12 represent the “primordial” repertoire of proteins which yielded several duplicons in humans. Although, both residues, T and S, have been found in active ATPases, the detailed catalytic consequences of their preference in most NLR proteins remains undefined.

**The Walker B** motif of ATPases, located in the nucleotide-binding site, is characterized by the conserved sequence pattern hhhhDD/E (h represents a hydrophobic amino acid). The proximal aspartate residue is crucial for coordinating binding of the Mg^2+^ cation, which has been shown to be required for nucleotide hydrolysis. The second acidic residue, usually glutamate, primes a water molecule for the hydrolysis of ATP [24].

Generally, the Walker A/P-loop and Walker B motifs are well conserved amongst ATPases (e.g. Apaf-1 and CED-4). However, our multiple sequence alignment revealed that all NLRs, with the exception of NAIP and NALP11, contain a modified Walker B motif, where the second acidic residue is missing. Thus, it remains elusive if NLRs harbouring these substituting amino acids (glycine, alanine or serine) within the Walker B motif, hhhhD[GAS]hDE, are still capable of nucleotide hydrolysis (Table 2, Figure 1). Interestingly, a recent publication by Ting *et al* reports that NALP12, which also contains a modified Walker B box, is capable of both, ATP hydrolysis and oligomerization [25]. Consequently, it is feasible to assume that NLRs use diverse mechanisms to prime the water molecule for ATP hydrolysis, where one might be the replacement of one conserved acidic amino acid by utilizing what we propose to term: an extended Walker B box. In many NLRs the extended Walker B box is composed of a conserved DE tandem motif that is located three residues downstream of the first D in the Walker B motif. Exceptions are CIITA, NALP4, NALP8, NALP13 with an EE, NALP5 with a DD, NOD5 with an EH sequence, Ipaf with NE and NALP11 with DN, respectively (Table 2). These data show that although observed for NALP12, the extent of ability and capacity to hydrolyse ATP may vary amongst NLR proteins based on their individual extended Walker B motifs. This is in line with findings that, in this respect, Apaf-1, CED4 [26] and DARK [27] are extremely different, too. Therefore, the here defined extended Walker B box represents a key element for the further investigation of distinct NLRs, their function and the involvement of ATP hydrolysis in their specific signaling pathways.

**The Sensor 1** motif is typically found adjacent to the Walker A and B motifs and interacts with or “senses” the γ-phosphate of ATP (Figure 3) [24]. In AAA+ family members this motif consists of a conserved arginine located right after β-strand 4, joined by two serine or threonine residues and further upstream by three hydrophobic residues. Within this sequence context, it has been suggested that arginine coordinates nucleotide hydrolysis and conformational changes between subunits [28]. Our analyses revealed that the Sensor 1 motif of Apaf-1 and all NLRs with the exception of Ipaf, NALP4, NALP9 and NALP13 contain this conserved arginine (Table 2). Moreover, we observed that except for NALP4 (AI), NALP8 (MI), and NALP9 (AL), the first two threonines are generally conserved in most NLRs.

**The Sensor 2** motif is a feature of AAA+ ATPases and is typically located in the region right after Sensor 1 before β-strand 5. This motif is usually characterized by a conserved arginine or lysine residue involved in nucleotide-binding and hydrolysis. We observed that this specific feature is generally missing in proteins belonging to the STAND class, or at least could not be functionally assigned based on their primary sequence. However, by analyzing the structure of Apaf-1 in its closed form, we observed that a unique feature comes to light. In comparison to other AAA+ ATPases, Apaf-1 displays the involvement of the WH domain in the coordination of ADP, instead of the missing Sensor 2 motif, with H438 and S422 contributing two hydrogen bonds to the coordination of the phosphate groups. Of particular interest in this case is H438, which can be regarded as replacement of the Sensor 2 motif, when compared to the structures of other AAA+ super family members [29]. Our structural alignments of NLR proteins with Apaf-1 reveal that Sensor 2 is also replaced by a conserved histidine in the WH domain of NLR proteins (Table 2, Figure 3). Importantly, we observed that the conserved histidine is part of a highly conserved sequence patch among NLR family members (Figure 1). This patch is characterized by the consensus sequence FxHxxQEhxA, which has been described as a unique feature of the NAIP-like subfamily among the STAND clade [19] and now points to a common involvement of this patch in NLRs acting in a Sensor 2-like manner. We observed that almost all NLRs harbour this conserved sequence with slight variations concerning the glutamate residue. Exceptions are NALP6, NALP8, NOD5, CIITA, and NAIP, where the conserved histidine is not present (Figure 1). It is not clear whether these NLRs replace the histidine by another feature or are incapable of ATP hydrolysis. As mentioned above, the Sensor 2 motif in AAA+ ATPases is composed of a conserved arginine residue that completes the active site of the neighbor molecule in the oligomer, where it is supposed to be involved in nucleotide-binding [24]. In fact, some NLR proteins such as NAIP, NALP2, NALP4, and NOD1 display an arginine residue downstream of the Sensor 1 motif that could function as a Sensor 2 motif. However, the conserved histidine residue present in the WH domain of NALP2, NALP4, and NOD1 may still be capable to substitute the function of Sensor 2.

### 1.4 Additional domains and motifs

The GxP signature is a conserved motif located in the small helical subdomain (C-domain) [18] at the C-terminal region of the NACHT domain (Figure 1) [19]. Interestingly, the conserved proline interacts with the adenine moiety of the bound ATP molecule (Figure 3). Our alignment analyses revealed that most NLRs display this highly conserved proline residue (with the exception of NAIP (T) and NALP 11 (A)), but lack the conserved glycine residue (Figure 1), suggesting a key feature assigned to the proline among NLRs. As described, additional domains following the NACHT domain are the WH domain, also referred to as HETHS domain [19] containing the conserved histidine motif, and the SH domain, which consists of eight alpha helices in a superhelical arrangement of yet unknown function.

Additional NLR sequence motifs are the cysteine rich region in the NACHT domain, containing a VCWxVCT motif located adjacent to the nucleotide-binding site (Figure 1), which plays a role in nucleotide recognition. Another feature is a highly conserved patch located in the WH domain of Apaf-1. This feature displays the sequence METEEV (Figure 1, Table 2) where the second glutamate is part of the interface to the adjoining CARD domain and forms hydrogen bonds to backbone atoms in the loop connecting helices 3 and 4 of the CARD domain. This interaction, which may lead to the stabilization of the dormant form seems to be conserved in the whole NLR family. In NLR proteins there is in place of the methionine a highly conserved phenylalanine residue (Figure 1, Table 2). Only NALP2, NALP8, and CIITA contain a leucine instead of the phenylalanine residue. Also the glutamate residues are conserved to a certain degree or substituted by an aspartate residue within NLR proteins. These conserved motifs are most likely involved in intra- and intermolecular interactions required for stabilization of the closed form and formation of the active signaling platform.

### 1.5 Features important for intermolecular interactions and oligomerization

**Figure 4:**
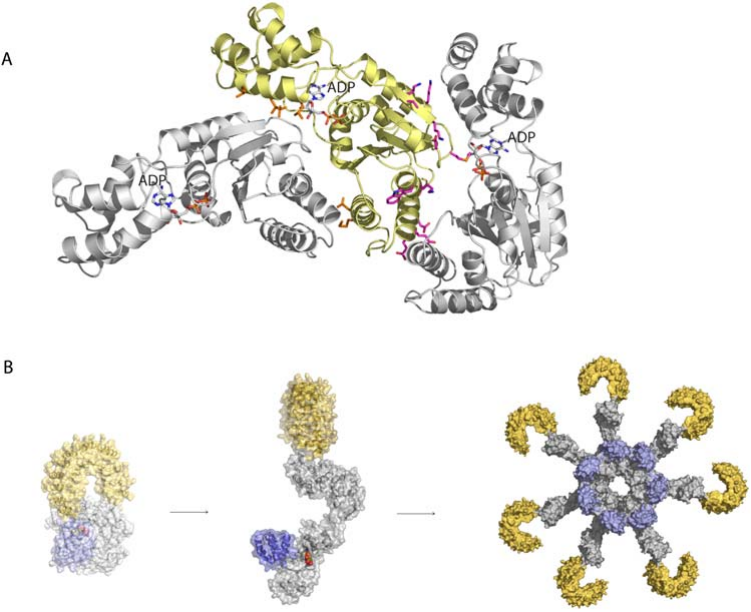
A NLR oligomerization interface: Apaf-1 oligomer modeled on the basis of the NtrC1 heptamer crystal structure. For clarity only three NACHT domains are shown in ribbon representation with an ADP molecule depicted in sticks to highlight the nucleotide-binding site in each domain. Side-chains of residues in the oligomerization interfaces are shown in sticks color-coded according to the alignment in Figure 1. The two interfaces form across the nucleotide-binding site of the NACHT domain including the GxP domain. B Model of NLR activation and inflammasome formation based on the Apaf-1 apoptosome.

Since our detailed sequence analyses revealed that most NLRs and Apaf-1 share the same domain architecture and many secondary structure features, the availability of structural and mechanistic data for Apaf-1 provides the opportunity to link conserved sequence features of NLRs to functional aspects of NLR signaling. Cytochrome c activated Apaf-1 has been shown to undergo an ATP-hydrolysis-dependent conformational rearrangement in order to form heptamers through an interaction of its NACHT domains. Interestingly, the heptamers were proposed to arrange in a ring-like structure, which is usually found in AAA+ ATPases such as RuvB or NtrC1 [30]–[32]. We consequently generated homology models for the NACHT-WH-SH regions and analyzed the distribution of conserved motifs and residues in order to deduce a putative mechanism for NLR oligomerization (Figure 4A). Although an alternative ring formation has also been proposed [33], we used the typical AAA+ like arrangement in which the interface is formed of surface residues in the NACHT domain (see orange and magenta boxes in Figures 2).

Based on the fact that all structural features required for oligomerization are present (Figure 4B), we hypothesize that NLRs are in principle capable of building signaling platforms like Apaf-1. This suggests that NLRs also use the ring-like arrangement of effector domains to recognize and activate signaling partners.

### 2. NLR effector domains and their corresponding binding partners

#### CARD-CARD interactions

Structural and mutational studies of the CARD domains of Apaf-1 and procaspase-9 have identified the essential motifs for procaspase-9 activation by Apaf-1 [34]. The interface of these two proteins has been shown to be mainly constituted by electrostatic interactions between an acidic and convex surface patch (helices 1 and 4) within the CARD domain of Apaf-1 and by a basic and concave surface patch (helices 2 and 3) within the CARD of procaspase-9. Among this homophilic CARD-CARD interaction, it has been shown that the crucial residues D27, E39, E40, and E41 are localized within the acidic region of Apaf-1 [35]. Furthermore, on the NLR protein NOD1, residues D42, D48, E53, D54, and E56 of the NOD1 CARD were suggested to mediate its interaction with its effector protein RICK. Complementary residues R444, K480, R483, R488 on the CARD domain of RICK were found in the putative interaction surface [35].

**Figure 5:**
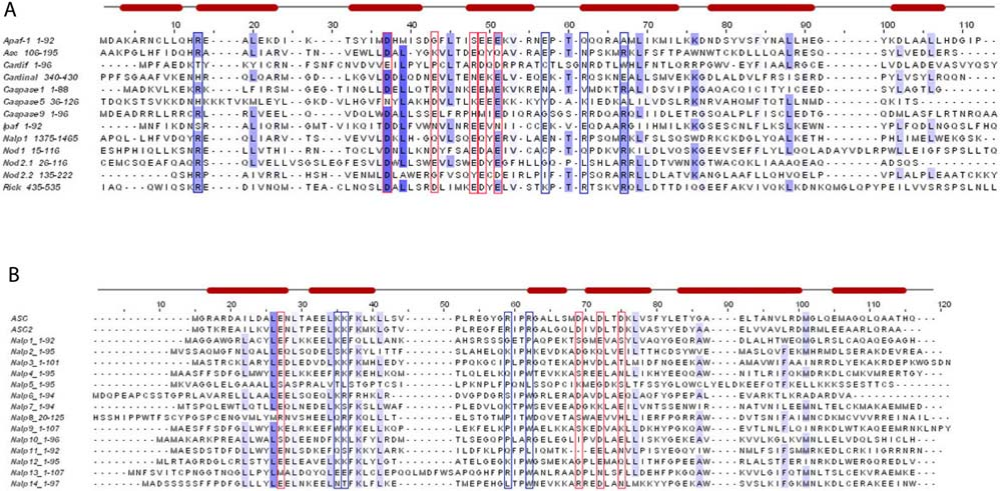
A Multiple sequence alignment of NLR and Apaf-1 CARD domains. Acidic key residues participating in the CARD-CARD interface are indicated by red borders. Residues that belong to the basic patch of the CARD-CARD interface are indicated by blue borders. Nod2.1 and Nod2.2 refer to NOD2 CARD domain 1 and 2, respectively. B Multiple sequence alignment of NLR PYRIN domains. Patch of negatively charged residues from ASC2 in helices 1 and 4 and their corresponding residues in the PYRIN domain containing NLR proteins (red box). Patch of positively charged residues from ASC2 in helices 2 and 3 and their corresponding residues in the PYRIN containing NLR proteins (blue box).

Based on these findings, we examined whether these residues, which are necessary for homophilic CARD-CARD interactions, are conserved among NLR CARDs and the CARDs of their effector proteins, respectively, by means of multiple sequence alignment (Figure 5A and 5B). Although the primary sequence conservation between CARD domains is generally low, we observed that the domains display a high degree of structural homology. Importantly the known interface residues of the homophilic CARD-CARD interactions of NOD1/RICK and Apaf-1/C9 are to a high degree conserved among NLR effector domains, caspases, and adaptor proteins (Table 3). Notably, the first and last residues of the acidic as well as the basic patch are highly conserved among the analyzed CARD domains, suggesting a pattern of interaction similar to the one described for Apaf-1/C9 or NOD1/RICK. These observations imply that the main principle of CARD-CARD interactions is based on the engagement of an acidic patch built of helices 1 and 4, with a basic patch composed of helices 2 and 3. However, the surrounding residues within this interface most likely define the specificity for interactions between CARD domains thereby ensuring the selectivity for the right interaction partner.

**Table 3:** Conservation of interface residues in the acidic patch and the basic patch based on the multiple sequence analysis in Figure 5A and 5B.

	Conserved residues Acidic patch					Basic patch			
**Apaf-1**	**D27**	S38	**E39**	**E40**	**E41**	R12	E46	Q49	A54
**ASC**	D134	E144	Q145	Y146	Q147	R15	E152	N155	R56
**Cardif**	E26	R37	D38	Q39	D40	T8	C46	N51	W56
**Cardinal**	D367	N378	E379	K380	E381	H352	E386	R389	E394
**Caspase 1**	D27	E38	E39	M40	E41	R10	N47	V50	R55
**Caspase 5**	N69	K80	E81	E82	E83	H48	Y88	K91	A96
**Caspase 9**	D27	H38	M39	I40	E41	**R13**	G47	R51	**R56**
**Ipaf**	D25	E36	E37	V38	N39	S8	E44	E47	R52
**NOD1**	D42	**E53**	**D54**	A55	**E56**	R27	C61	Q64	R69
**NOD2**	D58	E69	D70	Y71	E72	R38	G78	L81	R86
**RICK**	D461	E472	D473	Y474	E475	**R444**	**K480**	**R483**	**R488**

Bold residues contribute to the interface in NOD1/RICK and Apaf-1/Caspase9, respectively.

#### PYRIN-PYRIN interactions

To date, neither information from crystal structures nor mutational analysis of PYRIN domains or PYRIN-PYRIN interactions have been reported [36]. However, utilizing NMR, a recent report observed a highly bipolar organization of the human ASC and ASC2 PYRIN domains [36], [37], revealing that they resemble the molecular surface properties of CARD domains. These tertiary structure similarities between PYRIN and CARD domains indicate that like for CARD-CARD interactions, an electrostatic interface may play an important role for the biochemical properties and the interaction behavior of PYD-containing molecules [36], [37]. Based on this hypothesis, we propose that the already described interaction between the CARDs of Apaf-1 and caspase-9 can be utilized as a working model for PYRIN-PYRIN interactions as well. Following on this suggestion, one would expect that the residues in helices 2 and 3 of one PYD build an interface with the residues in helices 1 and 4 of a complementary PYD [37].

By utilizing multiple sequence alignments (Figure 5A and 5B) of both, CARD and PYD domains, we observed that the residues involved in the homophilic domain interfaces are conserved among the NLR family. However, mutational studies showed that these residues, which are important in a certain CARD-CARD interaction, are dispensable in the homotypic interaction of other proteins (e.g. the D42 mutant in Nod1 does not impair binding to Rick, but its corresponding residue in Apaf-1 is essential for its interaction with procaspase-9).

The proposed model of CARD-CARD and PYD-PYD interactions is that the acidic patch of one domain interacts with the basic patch of the other protein. Hydrophobic residues of adjacent regions are also suggested to be important in this interaction. Nevertheless, it is not clear so far, if there is a limited repertoire of structurally conserved motifs that may mediate interactions among death domain superfamily members. Therefore, more structural studies and mutational analysis of complexes built of those domains are necessary to define the motifs and interacting residues involved.

### 3. The LRR receptor domain

Similarly to the WD-40 repeats in Apaf-1, leucine-rich repeats are the ligand sensing motif of NLR proteins, a property they share with members of the TLR and RLR (RIG-I-like receptors) families. LRRs in general consist of 2–45 motifs of 20–30 amino acids in length and exhibit a typical curved horseshoe-like structure with a parallel beta sheet on the concave side and helical elements on the convex side [38].

In NLRs the C-terminal LRR domain is thought to act as a sensor of bacterial products. Yet, little is known about how the PAMP is interacting with the LRR or even how the LRR region interacts with the remainder of the NLR, since no structural data is available on these questions. Recently, some insight into the possible mechanism of ligand-receptor binding was provided by the two LRR-ligand complex structures of TLR1:TLR2 [39] and TLR4:MD2 [40]. Within the proposed LRR-ligand complex, the ligand-binding site is located at the concave surface of the LRR domain.

**Figure 6:**
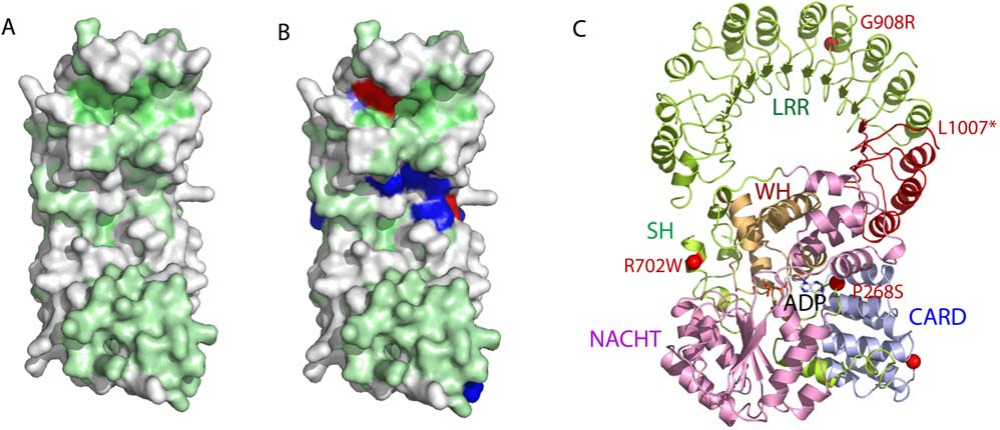
A Homology model of NOD2 LRR domain based on the ribonuclease inhibitor (pdb id: 1bnh). Predicted structure of NOD2 LRR domains with conserved residues shown in green and non-conserved residues in white. B Position of loss-of-function mutations shown in darkblue. Mutations found in CD patients are depicted in red. (Core forming residues that do not contribute to the ligand-binding patch are highlighted in lightblue). C NOD2 homology model based on templates Apaf-1 (aa1–581, PDB id: 1z6t, seqID 11%) and ribonuclease inhibitor (aa1–413, PDB entry: 1bnh, seqID 33%) (33). NOD2 model depicted as a cartoon color-coded according to domain structure: blue CARD2 aa95–182, green linker aa183–240, purple NACHT aa241–465, orange winged helix and superhelical domain aa466–734, green-yellow LRR aa735–1040). An ADP molecule bound in the ATPase active site is depicted in sticks. The position of the SNP mutations P268S, R702W, and G908R, are shown as red spheres. The truncation due to mutation L1007fsinsC is colored red.

In order to augment our understanding of the molecular mechanism of ligand recognition we generated a homology model of the NOD2 LRR domains based on the structure of the ribonuclease inhibitor (aa1–413, PDB id: 1bnh, seqID 33%) as a template. Additionally, we utilized the Consurf Server for the identification of functional regions in NLRs by surface mapping of phylogenetic information. Figure 6A shows the modeled LRR domains of NOD2 with highly conserved residues in the human NLR family colored in green and non-conserved residues shown in white. The figure clearly shows an extensive patch of conserved residues spanning the surface hinting to a function of these residues in signal sensing or the activation mechanism.

Our findings are in accordance with recently published work by Tanabe and colleagues, which showed loss of function mutations in the LRR domain to be located on the convex surface with additional residues on the concave region [41]. However, only those residues that are predicted to contribute to the convex surface are conserved in the corresponding regions of LRR proteins, whereas the residues on the concave surface are not (Figure 6A and 6B). On the other hand the loss-of-function mutations on the outer surface in the LRR domain do not form a continuous patch. They are scattered all over the molecule and are therefore not likely to form the ligand-binding site.

Our homology model suggests a putative ligand-binding pocket situated in the concave surface and supports earlier observations, where the predicted loss-of function-mutations W907L, V935M, E959K, C961Y, K989E, S991F as well as the Crohn's disease related mutation G908 have been mapped to the same area [41]. These amino acid residues do form a contiguous patch and therefore may point to the putative ligand-binding site (Figure 6B). Supporting this, is the fact that the location of this particular surface patch corresponds to ligand-binding sites in other LRR proteins [42]–[44]. Taken together, these results point to a common putative binding pocket located at the concave surface of the LRR, which, however, differs from protein to protein. Whether the patches on the convex surface do contribute to ligand-binding or eventually contribute to locking the NLR proteins in the dormant form remains to be further investigated.

#### Disease derived mutations of NLRs: implication on NLR function

Several diseases were found to arise from aberrant NLR function [45]–[49]. More accurately, they are caused by SNPs (single nucleotide polymorphisms) leading to point mutations in NLR genes. One particular intriguing SNP is SNP5 in NOD2 leading to Crohn's disease. To analyze the position of the SNP5 mutation P268S within NOD2, a NOD2 homology model was created based on templates Apaf-1 (aa1–581, PDB id: 1z6t, seqID 11%) and ribonuclease inhibitor (aa1–413, PDB id: 1bnh, seqID 33%). The SNP5 mutation P268S resides in the linker region before the first helix of the NACHT domain (Figure 6C). P268 constitutes part of the nucleotide-binding interface where it interacts with the adenine moiety of ADP. P268S disturbs the backbone conformation of the linker thus interferes with nucleotide binding and may alter the affinity and hydrolysis rate of the nucleotide-binding domain. Hence, SNP5 impairs the fine-tuned conformational states of the active-inactive balance of the NOD2 receptor and has therefore most likely a direct impact on its signaling properties.

In summary, our study clearly shows that the overall architecture and secondary structure features of most NLRs resemble those of Apaf-1. From the structural point of view, most of the NLR family members are therefore Apaf-1-like, with deviations including NOD2 (2 CARD domains), NALP1 (additional FIIND and CARD domain), NALP10 (missing LRR region), NOD5 and CIITA (undefined N-terminal region). Analyses of multiple sequence alignments revealed that all NLRs contain the crucial features for ATP-binding. In comparison to Apaf-1, most NLRs display a modified Walker B box. Since all NLRs, except for NAIP and NALP11, do not contain the crucial Walker B glutamate or aspartate required to activate the water molecule, they seem to have developed a new motif, the extended Walker B box to retain ATP hydrolysis activity. This is supported by the observation that NALP12 is able to bind and hydrolyze ATP [25]. Thus, our sequence analysis now provides the basis for further studies to elucidate whether the modified/extended Walker B box is functional.

Additionally, we identified one of the most intriguing features, which is the conserved histidine in the WH-domain, to be conserved among members of the NLR family. NLRs displaying this feature most likely assemble similar to Apaf-1 and activate their targets by oligomerization. Interestingly, NOD5, CIITA, NALP6, and NALP8 do not contain the conserved histidine in their WH domain. Whether their oligomerization mechanism and ATP hydrolysis capacity differ remains an open issue.

Our analyses of the effector domains of NLRs as well as those of their adaptors and target caspases, or kinases reveal a common interface, which is composed of charged surface patches. The presence of acidic and basic surface patches theoretically renders all CARD and PYD domains compatible for interaction with each other. Yet their distinct profile and that of surrounding residues found in the described interfaces ensure the specificity for each interaction. This selectivity allows a well-balanced fine-tuning of the elicited immune response.

Finally, sequence comparison of LRRs in human NLRs does not reveal one particular region that serves as the general ligand-binding site. This suggests that individual NLRs evolved highly specialized modes to recognize specific ligands. However, conserved residues found within this domain may contribute to the intramolecular interaction or backfolding of the LRR region in order to regulate NLR activation. Our results serve as a basis for further mutational and functional analyses required to more precisely define the role of LRRs in ligand recognition and NLR activation.

## Methods

### Sequence alignments

NLR protein sequences (see Table 1) were submitted to profile-sequence searches with the FFAS server (http://ffas.ljcrf.edu) [15]. Secondary structure prediction was done using the predictprotein server (http://www.predictprotein.org) [16]. We analyzed the human sequences for NACHT domain paralogues (about 410 residues). Multiple alignments were created using muscle [23] and m-coffee [50] with default options in the aforementioned sequences and the Apaf-1 sequence. The alignment was manually adjusted according to secondary structure prediction.

### Evolutionary analysis

The alignments were used to run phylogenetic probabilistic analyses using the parallel implementation of MrBayes [51]. The sequence of Apaf-1 was used to root the tree in all cases. A total number of 200000 generations were run in 4 independent chains. The model used to set the priors for amino acid data was an average of all the available models and a sample was obtained each 10 generations. Once convergence was reached, a total of a credible 6973 trees were sampled and clade credibility values (probabilities) calculated. In order to check how the paralogues arrange in a bigger tree, homologous sequences were retrieved from Uniprot databases from close organisms. The new sequences (31) were re-aligned to the original multiple alignment using T-coffee. To keep the clarity of the tree, we used a final number of 54. As in previous cases, 200000 generations were run. The frequency of sampling was each 10 generations. A total of credible 2777 trees were then sampled.

### Homology modeling

The protein structure with the highest scoring alignment from the FFAS-search was used as a template for modeling the structures with the SCWRL-Server (http://www1.jcsg.org/scripts/prod/scwrl) [52]. Models were evaluated using PROSA (https://prosa.services.came.sbg.ac.at/prosa.php) manually inspected, analyzed, and figures were prepared using Pymol (http://pymol.sourceforge.net). The degree of conservation was calculated using the ConSurf Server (http://consurf.tau.ac.il).
